# Cheong-sang-bang-pung-san alleviated hepatic lipid accumulation by regulating lipid metabolism *in vitro* and *in vivo*

**DOI:** 10.3389/fphar.2023.1223534

**Published:** 2023-09-06

**Authors:** Yun-Mi Kang, Kwang-Youn Kim, Tae In Kim, Yeon-Ji Kim, Han-Hae Kim, Kyungho Kim

**Affiliations:** ^1^ Korean Medicine (KM) Application Center, Korea Institute of Oriental Medicine, Daegu, Republic of Korea; ^2^ Korean Medicine Life Science, University of Science and Technology, Daejeon, Republic of Korea

**Keywords:** Cheong-sang-bang-pung-san, fatty liver disease, lipid accumulation, lipid metabolism, high-fat diet, HepG2 hepatocytes

## Abstract

**Introduction:** The occurrence of fatty liver disease, resulting from the accumulation of excessive fat within the liver, has been showing a significant and rapid increase. This study aimed to evaluate the therapeutic effects of Cheong-sang-bang-pung-san extract (CB) on fatty liver disease, and to elucidate the underlying mechanisms.

**Methods:** We used a high-fat diet (HFD)-fed fatty liver mice and free fatty acid (FFA) induced HepG2 cell lipid accumulation model. The levels of serum, hepatic, and intracellular lipid content were assessed. Histopathological staining was used to evaluate the extent of hepatic lipid accumulation. Real-time polymerase chain reaction and Western blotting were conducted to examine the expression of factors associated with lipid metabolism.

**Results:** We demonstrated that treatment with CB dramatically reduced body weight, liver weight, and fat mass, and improved the serum and hepatic lipid profiles in HFD-induced fatty liver mice. Additionally, CB alleviated lipid accumulation in HFD-fed mice by controlling lipid metabolism, including fatty acid uptake, triglyceride and cholesterol synthesis, and fatty acid oxidation, at the mRNA as well as protein levels. In free fatty acid-treated HepG2 cells, CB significantly reduced intracellular lipid accumulation by regulating lipid metabolism via the activation of AMP-activated protein kinase.

**Conclusion:** These findings provide insights into the mechanisms underlying CB’s effects on liver steatosis and position of CB as a potential therapeutic candidate for managing lipid metabolic disorders.

## 1 Introduction

Lipid metabolism is involved in several physiological and pathological pathways. The liver plays a key role as a metabolic hub for all lipid types (Nguyen et al., 2008). Lipid metabolism and homeostasis in hepatocytes are maintained through multiple mechanisms, including intracellular uptake, synthesis, storage, transport, consumption, and oxidative metabolism (Duan et al., 2022; Feng et al., 2023). Overnutrition enhances lipolysis within adipocytes, which leads to higher hepatic fatty acid levels and hepatic *de novo* lipogenesis. The excess fatty acid cannot undergo oxidative metabolism; instead, it is directed toward triglyceride (TG) synthesis, causing hepatic TG storage to increase and very-low-density lipoprotein to be overproduced (Alves-Bezerra and Cohen, 2017). Likewise, dysregulation of cholesterol homeostasis causes free cholesterol to accumulate in the liver, promoting the development of fatty liver disease and atherosclerosis (Li et al., 2021). In other words, fatty liver is associated with abnormalities of hepatic lipid metabolism that is a consequence of lipid acquisition pathways exceeding lipid disposal pathways (Ipsen et al., 2018).

The accumulation of excess lipid droplets within hepatocytes leads to hepatic steatosis in fatty liver, which is associated with an increased risk of metabolic diseases, such as obesity, and dyslipidemia, as well as chronic liver diseases, such as hepatitis, and cirrhosis. Some key risk factors and stages in pathogenesis are commonly shared by these diseases (Kotlyarov and Bulgakov, 2021). Therefore, preventing, and treating fatty liver disease is an important public health concern. However, the safety issues of clinically recommended lipid-lowering drugs have not yet been overcome (Mercep et al., 2022). Accordingly, there has been a steady demand for development of safe drug for treating dyslipidemia.

Traditional botanical medicine formulas have been shown to attenuate fatty liver disease and regulate hepatic lipid metabolism, thereby emerging as a new source of therapeutic agents for such diseases (Yang et al., 2019; Dang et al., 2020; Yan et al., 2020; Han et al., 2021). One of the traditional medicine prescriptions, Cheong-sang-bang-pung-san (“Qing-shang-fang-feng-san” in Chinese and “Seijo-bofu-san” in Japanese), is commonly used in clinical practice to treat purulent skin inflammations, such as acne and urticaria (Kim et al., 2019). This prescription was first mentioned in *Wanbinhuichun* and it is also mentioned in *Dongeuibogam* by Heo Jun of the Joseon Dynasty of Korea (Chen et al., 2016). Korea Food and Drug Administration has approved it as a clinical prescription, and it is most frequently administered to patients with acne vulgaris (Kim et al., 2016). The anti-inflammatory effects of Cheong-sang-bang-pung-san in the skin of patients with acne vulgaris (Chen et al., 2016) and urticaria (Yang et al., 2018) have been demonstrated. While traditional medicine holds promise as a potential source for new drug development targeting metabolic diseases, its impact on fatty liver disease remains unexplored.

This study aimed to investigate the regulatory effect of Cheong-sang-bang-pung-san extract (CB) in animal and cellular models of hepatic steatosis. We found that CB prevented high-fat diet (HFD)-induced fatty liver disease by regulating lipid metabolism. This study is meaningful in that it proposed the alternative therapeutic applications for dyslipidemia and expanded new indication of botanical medicine that is generally considered to be safe and effective agents.

## 2 Materials and methods

### 2.1 Preparation of CB extract

The botanical medicine materials were purchased from Omniherb Co. LTD. (Yeongcheon, Korea). Assurance of quality control for all the materials was validated according to the Korean Botanical Pharmacopoeia (Korea Food and Drug Administration, 2002). CB-containing botanical medicines and the ratios were listed in Table 1. The materials of CB were placed in distilled water at a ratio of 1:10 and heated at 115°C in the extractor (Gyeongseo Extractor Cosmos-600, Inchon, Korea) for 2 h. The resulting extract was filtered using a standard test sieve (150 μm) (Retsch, Hann, Germany) and freeze-dried to yield 25.9%. The lyophilized powder was stored at −80°C and dissolved in phosphate-buffered saline (PBS) before use.

**TABLE 1:** The crude metabolites of CB.

Scientific name	Latin name	Family	Active metabolites/markers	Amount (g)
***Saposhnikovia divaricata* Schischkin**	Saposhnikoviae Radix	Umbelliferae	cimifugin*, prim*-*O*-glucosylcimifugin	2.75
***Angelica dahurica* Bentham et Hooker f.**	Angelicae Dahuricae Radix	Umbelliferae	forsythol, oleanic acid, arctiin	3.00
**Forsythia suspensa Vahl**	Forsythiae Fructus	Oleaceae	arctigenin, arctiin	3.00
***Platycodon grandiflorum* A. De Candolle**	Platycodonis Radix	Campanulaceae	platycodin A-D, Betulin, Inulin	3.00
***Scutellaria baicalensis* Georgi**	Scutellariae Radix	Labiatae	baicalin, wogonin baicalein	2.62
***Cnidium officinale* Makino**	Cnidii Rhizoma	Umbelliferae	ferulic acid	2.62
***Schizonepeta tenuifolia* Briquet**	Schizonepetae Spica	Labiatae	Pulegone, limonene, schizonepetoside C	1.87
**Gardenia jasminoides Ellis**	Gardeniae Fructus	Rubiaceae	geniposide, genipin, crocin	1.87
***Coptis japonica* Makino**	Coptidis Rhizoma	Ranunculaceae	coptisine, berberine	1.87
***Citrus aurantium* Linné**	Aurantii Fructus Immaturus	Rutaceae	naringin, hesperidin	1.87
***Mentha arvensis* Linné var. piperascens Malinvaud ex Holmes**	Menthae Herba	Labiatae	menthol, menthone, camphene	1.87
***Glycyrrhiza uralensis* Fischer**	Glycyrrhizae Radix et Rhizoma	Leguminosae	glycyrrhizin, liquiritin, liquiritigenin	1.12

Assurance of quality control for all the materials was validated according to The Korean Herbal Pharmacopoeia (2022, Ministry of Food and Drug Safety, Republic of Korea).

### 2.2 Chemicals and reagents

Dulbecco’s modified Eagle’s medium (DMEM), fetal bovine serum (FBS), and penicillin streptomycin were purchased from HyClone (South Logan, UT, United States). Primary antibodies against sterol regulatory-element binding protein (SREBP)-1, α-tubulin, and β-actin antibodies were purchased from Santa Cruz Biotechnology, Inc (Santa Cruz, CA, United States). Primary antibodies against acetyl-CoA carboxylase (ACC), p-ACC, fatty acid synthase (FAS), carnitine palmitoyltransferase (CPT)-1α, AMP-activated protein kinase (AMPK) and p-AMPK were purchased from Cell Signaling Technology, Inc (Danvers, MA, United States). SREBP2, proprotein convertase subtilisin/kexin type 9 (PCSK9), and LDL receptor (LDLR) antibodies were purchased from Invitrogen (Waltham, MA, United States). Horseradish peroxidase-conjugated secondary antibodies were obtained from Jackson Immuno Research Laboratories, Inc. (West Grove, PA, United States). THUNDERBIRD SYBR qPCR Mix was purchased from TOYOBO (Kita-ku, Osaka, Japan). Oligonucleotide primers for ACC 1/2, liver X receptor (LXR) α, SREBP 1/2, FAS, hydroxy-3-methylglutaryl-coenzyme A reductase (HMGCoR), CPT-1α, LDLR, cluster of differentiation 36 (CD36), fatty-acid-binding protein (FABP), scavenger receptor class B type 1 (SR-B1) and glyceraldehyde-3-phosphate dehydrogenase (GAPDH) were purchased from Cosmogenetech (Seoul, Republic of Korea). To simultaneous analyzed CB, we obtained several metabolites according references that reported bioactivity and marker metabolites of CB. The reference standard compounds for simultaneous analysis, Baicalin and Baicalein, were purchased from sigma-aldrich (St. Louis, MO, United States). Wogonoside was obtained from Shanghai Sunny Biotech (Xiangyin Rd, Shanghai, China), Geniposide was provided by Wako (Chuo-ku, Osaka, Japan) and Hesperidin was purchased from Biopurify (Chengdu, Sichuan, China). All solvents for analysis, tertiary distilled water, methanol, acetonitrile, were HPLC grade obtained Merck (Darmstadt, Germany). Formic acid was purchased sigma-aldrich (St. Louis, MO, United States).

### 2.3 Animal experiments

Male fifty C57BL/6J mice (6-week-old, 20–24 g) were purchased from DooYeol Biothech (Seoul, South Korea). Mice were acclimatized under constant conditions (humidity, 50%–55%; temperature, 22°C ± 2°C; 12 h light/dark cycle). After 1 week of adaptation to the feeding conditions, mice were randomly separated into 5 groups of 10 each: (1) normal diet (ND) group, (2) HFD (40% fat diet, Research diet, D12109) group, (3) HFD treated with atorvastatin (10 mg/kg) group, (4) HFD treated with CB (100 mg/kg) group, and (5) HFD treated with CB (200 mg/kg) group. With the exception of the ND group, all mice were fed an HFD. CB was dissolved in PBS and orally administered on a daily basis for the last 9 weeks along with HFD in the 100 or 200 mg/kg CB groups. Body weights were measured once weekly. After 14 weeks of experiment, the animals were fasted overnight and sacrificed. Blood samples were collected from mice via cardiac puncture under 2%–2.5% isoflurane anesthesia. After cervical dislocation, the liver tissues, and gastrocnemius muscle were excised, rinsed with PBS, weighed and directly stored at −80°C until analysis. All part of animal experiments were conducted in accordance with the Care and Use of Laboratory Animals of the National Institutes of Health of Korea and were approved by the Institutional Animal Care and Use Committee of Korea Institute of Oriental Medicine (KIOM) (approval number KIOM-D-22-010).

### 2.4 Serum and hepatic biochemical parameters analysis

Blood samples were collected from each mouse and centrifuged at 1700 *g* for 15 min at room temperature to obtain serum samples. The samples were stored at −70°C until use. Serum or liver homogenate levels of TG, total cholesterol (TC), low density lipoprotein cholesterol (LDL-c), high density lipoprotein cholesterol (HDL-c), alanine aminotransferase (ALT), and aspartate transaminase (AST) were determined by enzymatic methods using commercial kits.

### 2.5 Histological analysis of liver

Collected liver tissues were fixed with 10% formalin. Paraffin-embedded tissue was cut into 3 μm sections and stained with hematoxylin and eosin (H&E). The stained sections were observed under a Leica DM IL LED microscope (Leica, Wetzlar, Germany).

### 2.6 qRT-PCR analysis

To isolate total RNA from the liver tissues or cells, TRIzol Reagent (Invitrogen, Waltham, MA, United States) was used according to the manufacturer’s protocol. Total RNA was quantified using an NanoDrop spectrophotometers (Thermo scientific, Waltham, MA, United States). Complementary DNA was synthesized from isolated total RNA (2 μg), oligo d(T)16 primer, and Avian Myeloblastosis Virus reverse transcriptase (AMV RT) with genomic DNA remover. Relative gene expressions of target gene versus reference gene were quantified using RT-qPCR analysis (CFX384 Touch Real-Time PCR Detection System; Bio-Rad, Hercules, CA, United States) with SYBR Premix. For calculate changes in gene expression as a relative fold difference, the comparative quantification cycle method was used. The Cq (Target gene–reference gene) values of target genes ACC1/2, LXRα, SREBP1/2, HMGCoR, CPT-1α, LDLR, CD36, and FABP were normalized to that of GAPDH.

### 2.7 Western blotting assay

Total protein was extracted from liver tissues or cells using PRO-PREP™ protein extraction kit (Intron Biotechnology, Seoul, Republic of Korea). The protein concentration was measured using the protein assay reagent (Bio-Rad, Hercules, CA, United States). Proteins were separated by 10%–12% SDS-PAGE, and transferred to polyvinylidene difluoride membrane. The membrane was blocked for 1 h with 5% BSA followed by incubation with primary antibodies at 4°C overnight. After washing three times with Tween 20/Tris-buffered saline (T/TBS), the membranes were incubated with a horseradish peroxidase-conjugated secondary antibody (dilution, 1:2500) for 2 h at room temperature. The blots were again washed three times with T/TBS, and then bands were detected via enhanced chemiluminescence reagent (Bio-Rad).

### 2.8 Cell culture and treatment

Human hepatic cell line HepG2 was obtained from the Korean Cell Line Bank (KCLB, Seoul, Republic of Korea). HepG2 cells were grown in DMEM supplemented 10% FBS and 1% penicillin/streptomycin at 37°C under a humidified atmosphere of 5% CO_2_. The cells were seeded at a density of 2× 10^5^ cells per well into 6-well plate and then treated with 0.5 mM oleic acid (OA; O7501, Sigma-Aldrich) and 0.25 mM palmitic acid (PA; P0500, Sigma-Aldrich) dissolved in culture medium containing 10% BSA (A1595, Sigma-Aldrich) with or without different concentrations of CB (50, 100, and 200 μg/mL) for 24 h. Control cells were treated with 10% BSA only.

### 2.9 Cell viability assay

The cell viability was assessed using MTT assy. In brief, HepG2 cells were plated into a 96-well plate with concentration of 1×10^5^ cells per well. After incubation for 24 h, the cells were treated with different concentrations of CB (50, 100, and 200 μg/mL) for 24 h. Afterwards, the cells were added with MTT working solution (5 mg/mL) and incubated for another 4 h. The supernatant from the plates was removed, and the purple formazan product was dissolved in DMSO. The absorbance was measured on an Epoch microplate spectrometer (Biotek, Winooski, VT, United States) at 540 nm.

### 2.10 Measurement of lipid accumulation oil red O staining

After stimulation with OA + PA mixture, the cells were washed with PBS and fixed with 10% formalin in PBS for 1 h, followed by staining with Oil Red O working solution (3 mg/mL in 60% isopropanol) for 2 h. The cells were rinsed with distilled water and observed under a microscope. Next, the Oil Red O dye was extracted with isopropanol to determine the intracellular lipid content and was assessed using an Epoch microplate spectrometer at 520 nm.

### 2.11 HPLC-DAD instrument and conditions to quantify analysis

The CB was dissolved in water at 10 mg/mL concentration using ultrasonicator (JAC Ultrasonic JAC-3010) for 30 min. After dissolved, CB solution was filtered through a 0.2 μm PVDF membrane and then 10 μL of filtrate was injected to HPLC analysis. Stock solution of single compounds (Baicalin, Baicalein, Wogonoside, Hesperidin, and Geniposide) were prepared at 1000 ppm with HPLC grade methanol and serially diluted at standard curve concentration of each. Standard stock solution was used for quantitative analysis after 0.2 μm PETE membrane filtration. Simultaneous determination of five metabolites (Baicalin, Baiclein, Wogonoside, Hesperidin, Geniposide) were conducted using using Dionex Ultimate 3000 system formed with column oven, an auto sampler, a binary pump and diode assay UV/VIS detector (DAD; Dionex Corp., Sunnyvale, CA, United States). HPLC condition were follows; column was Waters X bridge (250 mm × 4.6 mm, 5 μm) connected to a C18 guard cartridge (4.0 mm × 3.0 mm), column temperature and auto sampler were kept 40°C and 20°C respectively, eluted condition was flow rate 1.0 mL/min with gradient of 0.1% formic acid (solvent A) and 0.1% formic acid acetonitrile (solvent B), solvent gradient method was applied; 0–40 min, 10–60%B; 40–50 min, 60–95%B; 50–55 min. 95%B, chromatogram detected under 280 nm and 245 nm. Calibration curves, assessed by standard solution and the limits of detection (LOD) and quantification (LOQ) under the chromatographic conditions, were determined by injecting a series of standard solutions. Each sample was three injected under same condition and data was processed using Chromeleon 7 software (Thermo Fisher, Counteaboeuf, France)

### 2.12 Statistical analysis

Statistical analysis was performed using GraphPad Prism 5 software (GraphPad software, San Diego, CA, United States). Data are presented as the mean ± standard deviation (SD) of triplicate experiments. Statistical significance was determined using ANOVA and Dunnett’s *post hoc* test, and *p*-values of less than 0.05 were considered statistically significant.

## 3 Results

### 3.1 CB improved HFD-induced obesity phenotypes and serum lipid levels

**FIGURE 1:**
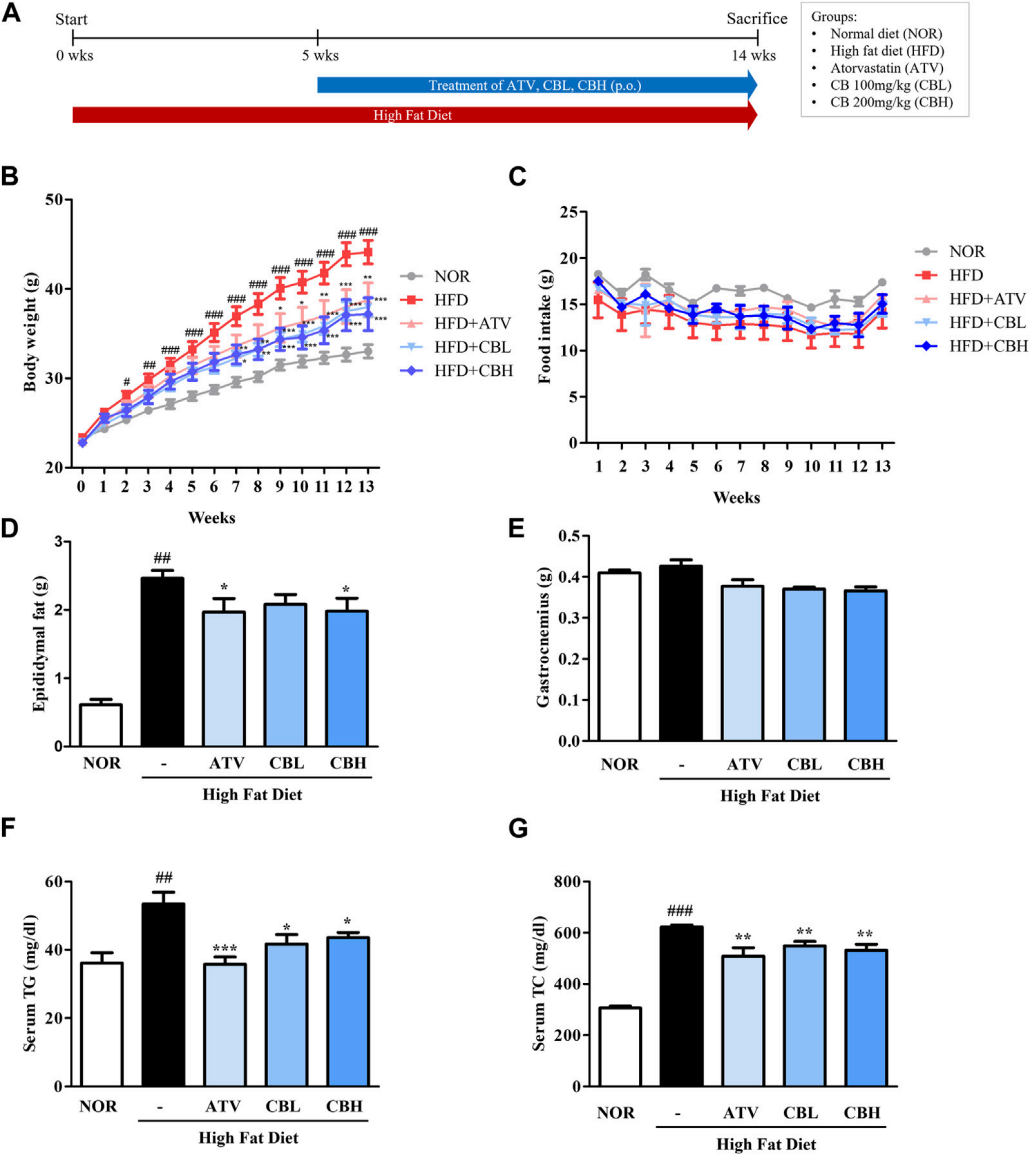
The effect of CB on obesity phenotypes and biochemical parameters in HFD-induced fatty liver mice. **(A)** Experimental scheme of the *in vivo* study. **(B)** Body weight and **(C)** food intake during 14 weeks in HFD-induced fatty liver mice. **(D)** Epididymal fat pad and **(E)** gastrocnemius weight in HFD-induced fatty liver mice. Serum levels of **(F)** triglycerides and **(G)** total cholesterol in HFD-induced fatty liver mice. Data represent means ± standard deviation (SD) (*n* = 10). #*p* < 0.05, ##*p* < 0.01, ###*p* < 0.001 vs. the control group; *p < 0.05, **p < 0.01, ***p < 0.001 vs. the HFD group. CB, Cheong-sang-bang-pung-san extract; HFD, high-fat diet; SD, standard deviation.

The *in vivo* experimental scheme is described in Figure 1A. Body weight and the amount of epididymal white adipose tissue were markedly increased in mice fed a HFD for 14 weeks compared with control mice (Figures 1B, D). However, CB treatment significantly attenuated both these phenotypes. The food intake (Figure 1C) or muscle (gastrocnemius) mass (Figure 1E) did not differ significantly between the groups. In addition, the serum levels of TG and total cholesterol were elevated in HFD-fed mice compared with control mice, while CB treatment significantly reduced their levels in HFD-fed mice (Figures 1F, G). These results suggest that CB alleviated HFD-induced obesity phenotypes and abnormal lipid changes.

### 3.2 CB ameliorated HFD-induced hepatic steatosis and liver injury

**FIGURE 2:**
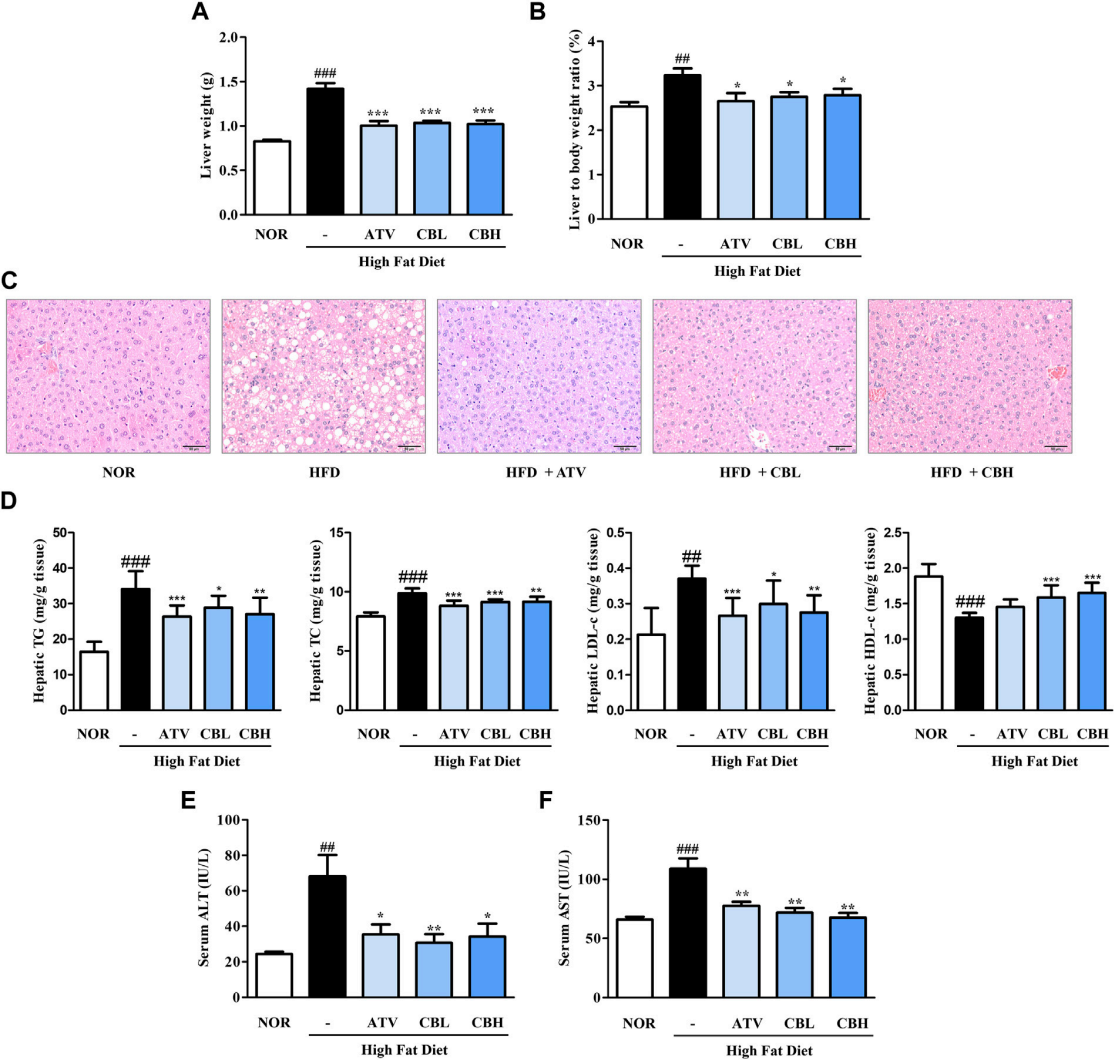
The effect of CB on hepatic phenotypes and biochemical parameters in HFD-induced fatty liver mice. **(A)** Liver weight and **(B)** weight ratio to the body weight of mice. **(C)** Representative histological images of the liver tissue were assessed using H&E staining (200×). Hepatic levels of **(D)** triglycerides, total cholesterol, LDL-cholesterol, and HDL-cholesterol in HFD-induced fatty liver mice. Serum levels of **(E)** ALT and **(F)** AST in HFD-induced fatty liver mice. Data represent means ± SD (*n* = 10). ##*p* < 0.01, ###*p* < 0.001 vs. the control group; *p < 0.05, **p < 0.01, ***p < 0.001 vs. the HFD group. H&E, hematoxylin and eosin; LDL, low-density lipoprotein; HDL, high-density lipoprotein; ALT, alanine transaminase; AST, aspartate transaminase.

Lipid metabolism and liver function are closely related. Therefore, we investigated whether CB treatment could inhibit hepatic steatosis and improve hepatic function. Liver weight significantly increased in the HFD group, but this increase was markedly blunted in the CB-treated groups (Figures 2A, B). Furthermore, H&E staining showed that lipid droplets were more numerous and larger in the HFD group, while CB treatment reduced both their number and their size (Figure 2C). Consistent with hepatic lipid accumulation, the hepatic levels of total cholesterol, TG, and low-density lipoprotein-cholesterol were also higher in HFD-fed mice than in control mice, whereas CB treatment significantly decreased these lipid levels. Conversely, CB treatment reversed the HFD-induced decrease in the level of high-density lipoprotein-cholesterol in the livers of mice (Figure 2D). Additionally, CB treatment significantly attenuated the HFD-induced increase in the levels of serum alanine and aspartate transaminases (Figures 2E, F), indicating that CB induced body weight loss and inhibited lipid accumulation in HFD-fed mice without resulting in toxicity.

### 3.3 CB regulated lipid metabolism in HFD-induced hepatic steatosis

**FIGURE 3:**
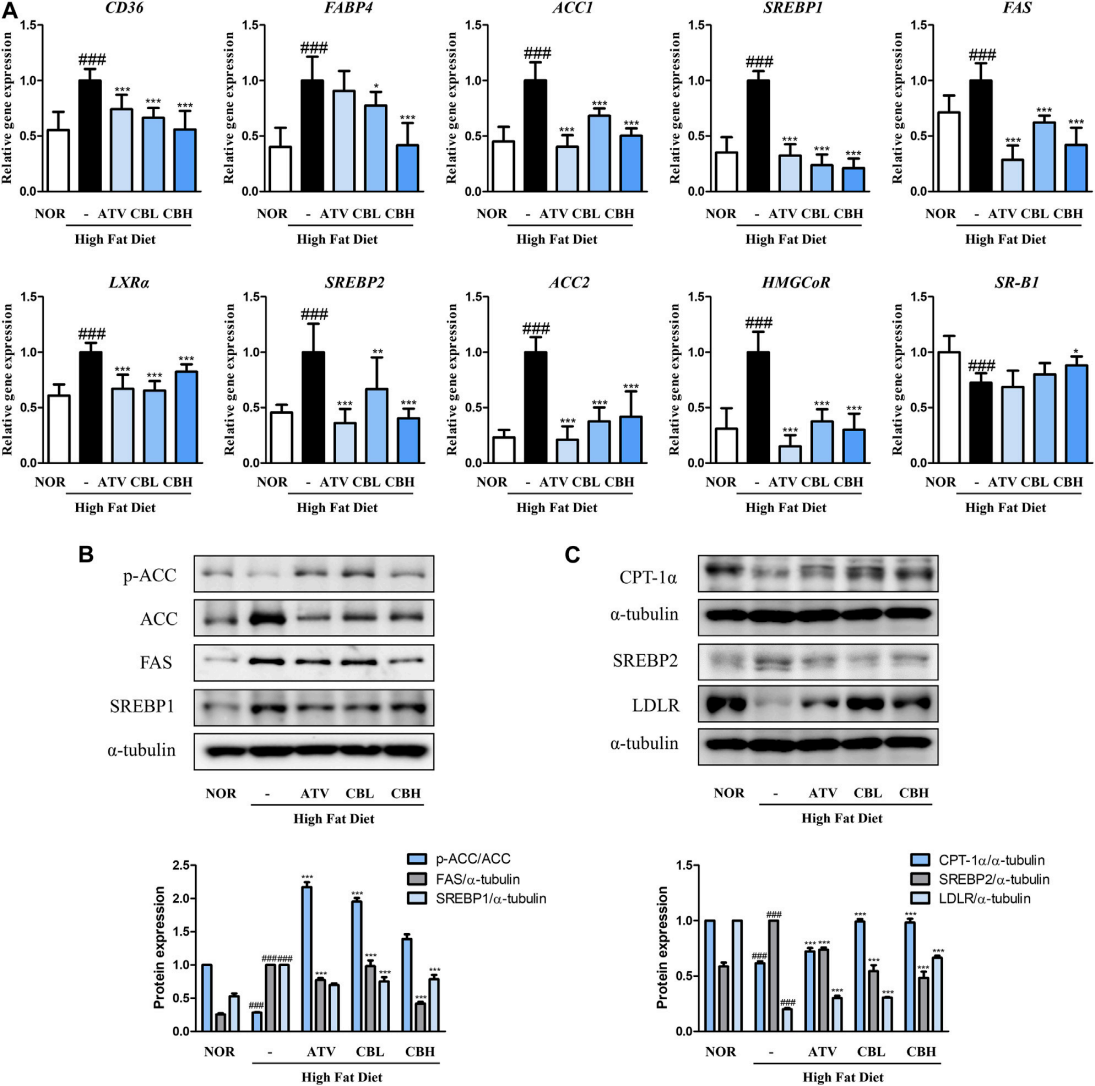
The effect of CB on the expression of markers related to lipid metabolism in HFD-induced fatty liver mice. The mRNA expressions of **(A)** *CD36*, *FABP4*, *ACC1*, *SREBP1*, *FAS*, *LXRα*, *SREBP2*, *ACC2*, *HMGCoR*, and *SR-B1* were quantified by qRT-PCR and normalized to GAPDH control **(B)** p-ACC, ACC, FAS, SREBP1, **(C)** CPT1α, SREBP2, and LDLR protein levels were determined by Western blotting, and normalized to α-tubulin expression. Densitometric analysis was performed using ImageJ. Data represent means ± SD (*n* = 10). ###*p* < 0.001 vs. the control group; *p < 0.05, **p < 0.01, ***p < 0.001 vs. the HFD group.

To better understand how CB inhibited lipid accumulation in the liver, the mRNA expression of genes involved in fatty acid synthesis and cholesterol metabolism was examined using quantitative reverse-transcription PCR. Compared with the control group, the HFD group showed significant upregulation of the lipid uptake markers *CD36* and *FABP4*, the lipogenesis markers *ACC1*, *SREBP1*, *FAS*, and *LXRα*, and the cholesterol synthesis markers *SREBP2*, *ACC2*, and *HMGCoR* (Figure 3A). On the other hand, *SR-B1*, a marker of cholesterol efflux, was downregulated. CB treatment reversed all these gene expression trends. Next, we determined how CB affected hepatic signaling using Western blotting. CB significantly enhanced the protein levels of phospho-ACC, CPT1α, and low-density lipoprotein receptor while reducing those of FAS, SREBP1, and SREBP2 in the liver of HFD-fed mice (Figures 3B, C). These results suggest that CB treatment altered multiple hepatic gene pathways involved in fatty acid, TG, and cholesterol metabolism in HFD-induced fatty liver mice.

### 3.4 CB ameliorated free fatty acid (FFA)-induced lipid accumulation in HepG2 hepatocytes

**FIGURE 4:**
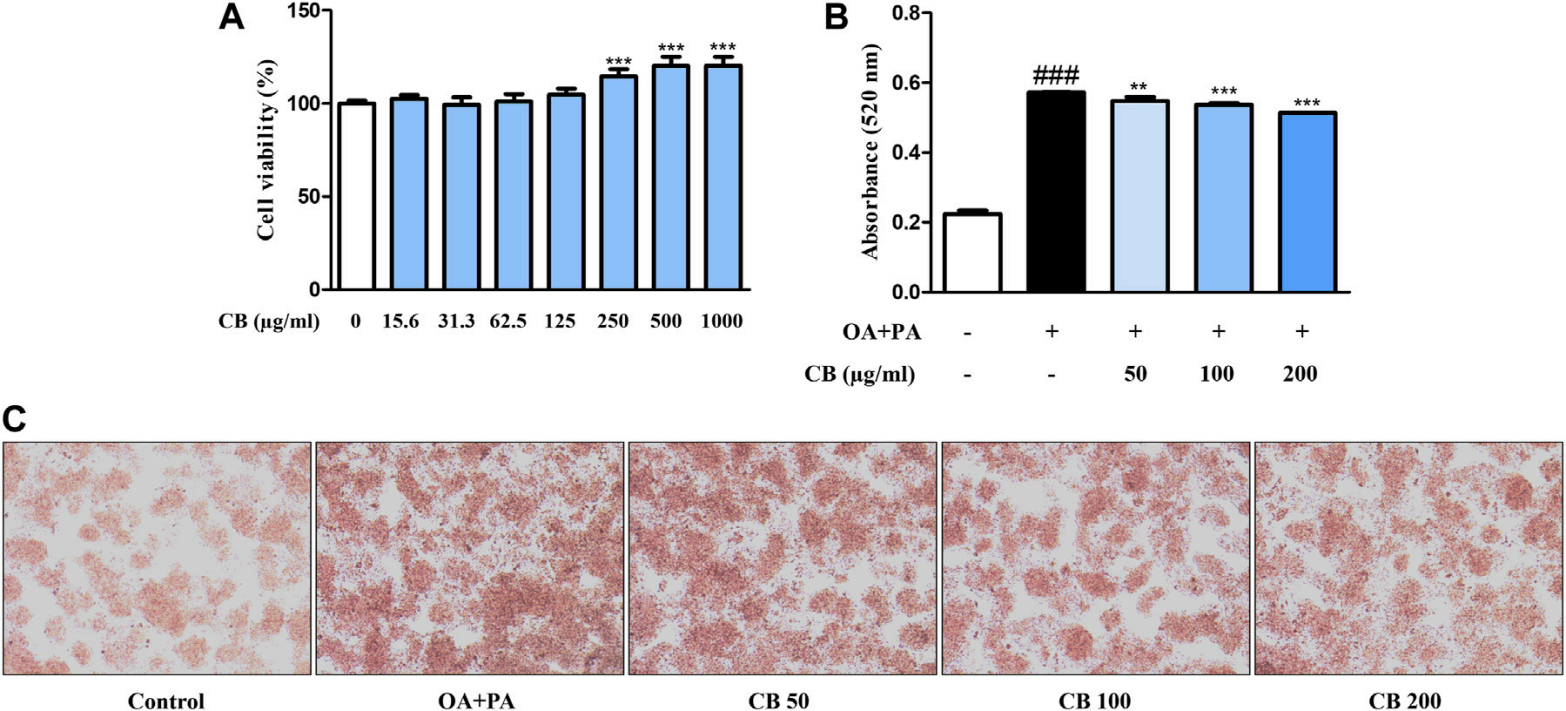
The effect of CB on lipid accumulation in FFA-induced HepG2 hepatocyte steatosis. **(A)** Cell viability was determined using the MTT assay. ***p < 0.001 vs. untreated cells. **(B)** Lipid accumulation was measured using Oil red O staining. Quantitative content was measured at 520 nm. **(C)** Oil Red O-stained image of HepG2 cells observed under a microscope (200×). Data represent means ± SD. ###*p* < 0.001 vs. the control group; **p < 0.01, ***p < 0.001 vs. the FFA-treated group.

HepG2 is most commonly used in liver metabolism and hepatotoxicity studies (Donato et al., 2015). To further assess the role of CB in an *in vitro* human cell system, we used a model of HepG2 cells. To determine the cytotoxicity of CB, cell viability was measured using the MTT assay. A range of 15.6–1000 μg/mL of CB was found to be non-cytotoxic to HepG2 cells (Figure 4A). Therefore, 50, 100, and 200 μg/mL of CB were used in subsequent experiments. The effect of CB on lipid accumulation in HepG2 cells was assessed using Oil red O staining. FFA treatment significantly increased the intracellular lipid level in HepG2 hepatocytes, whereas CB significantly suppressed lipid accumulation (Figures 4B, C). These results suggest that CB exhibited inhibiting effect on hepatic lipid accumulation in human liver cell without any cytotoxicity.

### 3.5 CB regulated lipid metabolism via the AMPK pathway in FFA-stimulated HepG2 hepatocytes

**FIGURE 5:**
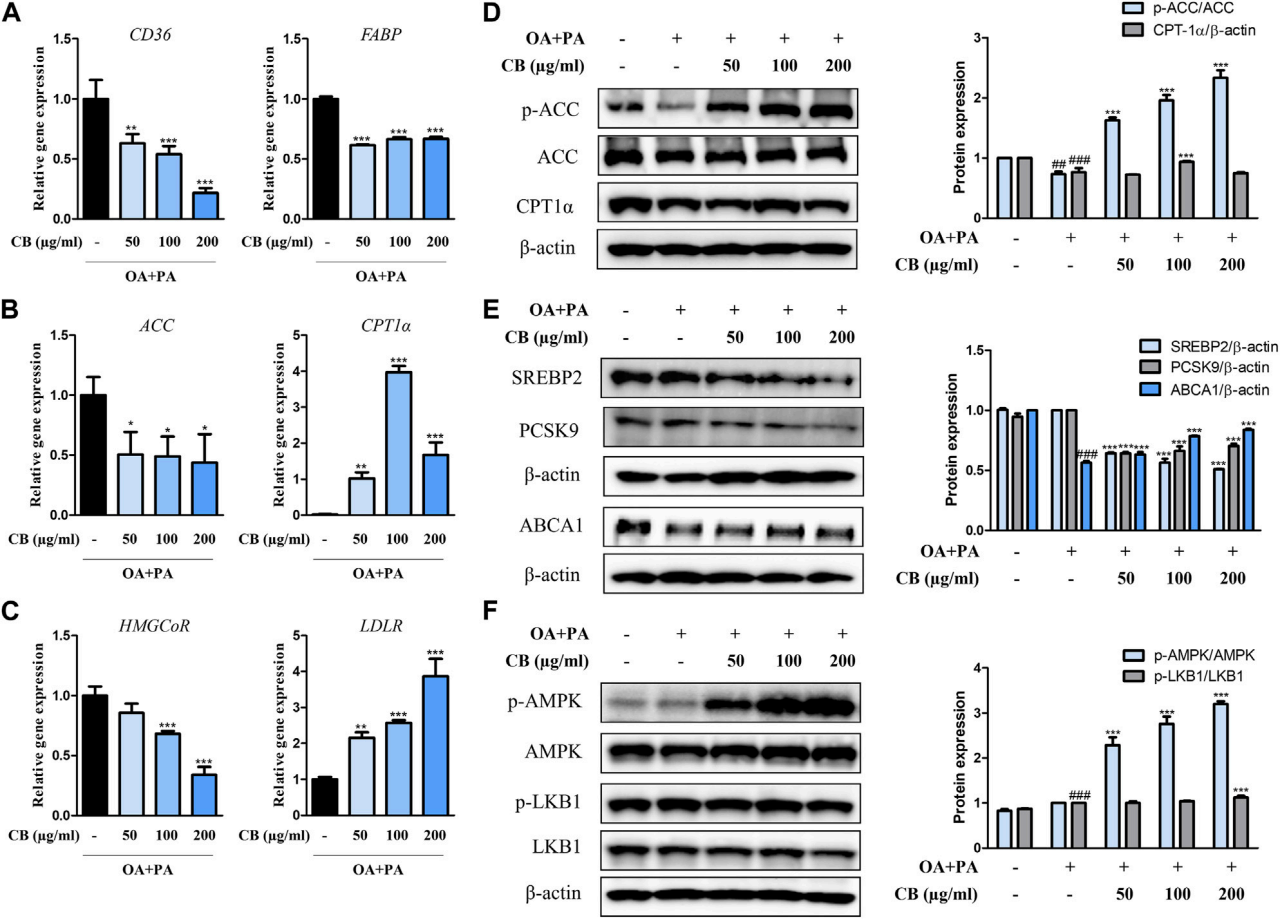
The effect of CB on the expression of markers related to lipid metabolism in FFA-induced HepG2 steatosis. The mRNA expression of **(A)** *CD36*, *FABP*, **(B)** *ACC*, *CPT1α*, **(C)** *HMGCoR*, and *LDLR* were quantified by qRT-PCR and normalized to GAPDH control. **(D)** p-ACC, ACC, CPT1α, **(E)** SREBP2, PCSK9, ABCA1, **(F)** p-AMPK, AMPK, p-LKB1, and LKB1 protein levels were determined by Western blotting, and normalized to β-actin expression. Densitometric analysis was performed using ImageJ. Data represent means ± SD. ##*p* < 0.01, ###*p* < 0.001 vs. the control group; *p < 0.05, **p < 0.01, ***p < 0.001 vs. the FFA-treated group.

To investigate the inhibitory mechanism of CB in human cells, the expression levels of relevant genes were examined. Compared with the FFA-treated group, *CD36*, *FABP*, *ACC*, and *HMGCoR* were downregulated in the CB-treated group, whereas *CPT1α* and *LDLR* were upregulated (Figures 5A–C). FFA treatment remarkably decreased the hepatic expression of phospho-ACC compared with untreated cells, but CB treatment elevated its levels in a dose-dependent manner (Figure 5D). The expression of CPT1α, a fatty acid oxidation marker, was higher in CB-treated cells (100 μg/mL) than in FFA-treated cells (Figure 5D). In addition, administering CB to FFA-treated cells dramatically decreased the expression of SREBP2 and PCSK9, which are related to cholesterol metabolism. CB also reversed the decrease in ATP-binding cassette transporter A1 expression induced by FFA (Figure 5E). Compared with the control and stimulated groups, AMPK phosphorylation markedly increased after CB treatment, while the expression of total AMPK remained unchanged (Figure 5F). The levels of phosphorylated liver kinase B1 did not differ significantly between the groups (Figure 5F). These results indicate that CB might prevent lipid accumulation in liver by controlling the expression of factors that are involved in lipid metabolism in human *in vitro* system.

### 3.6 Simultaneous quantitation of marker metabolites in CB

**FIGURE 6:**
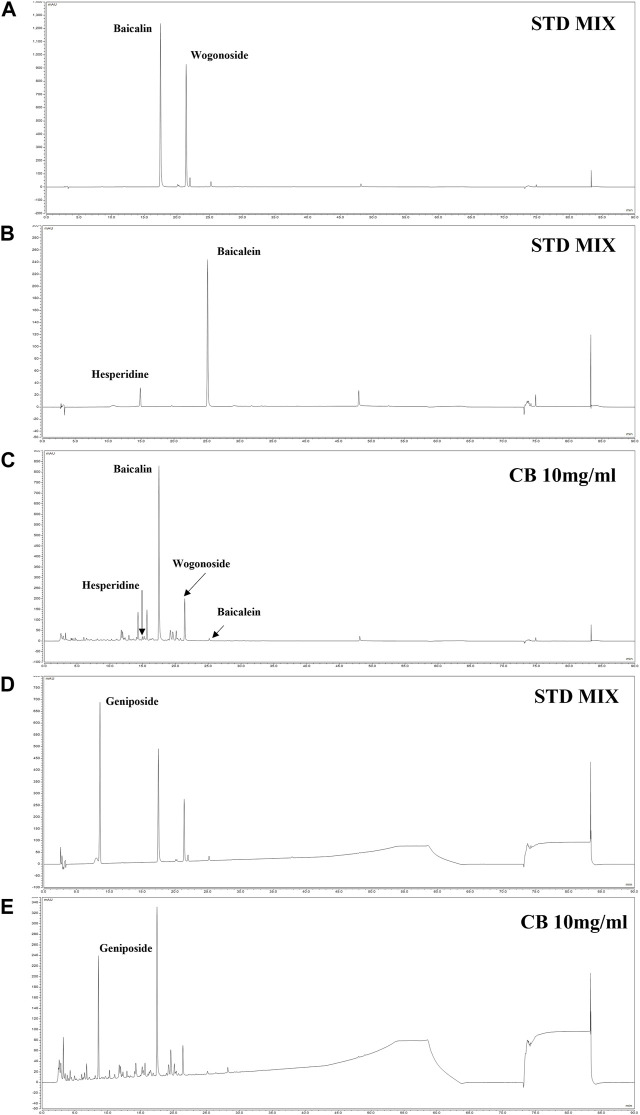
HPLC chromatogram of CB. HPLC-DAD chromatogram of standard compound mixture **(A, B)** and CB 10 mg/mL at 280 nm **(C)**. The standard compound geniposide 200 ppm **(D)**; and CB 10 mg/mL **(E)** at 245 nm. The peaks are Geniposide (8.537 min), Hespiridine (14.940 min), Baicalin (17.467 min), Wogonoside (21.397 min) and Baicalein (25.140 min).

The simultaneous analysis method was developed in the present study was efficiently applied to the quantitative analysis of five marker metabolites in CB extract. The marker metabolites were detected on HPLC analysis method that we developed and determined the contents of each metabolite in CB extract. The content of Baicalin, Baicalein, Wogonoside, Hesperidin and Geniposide were 11.29 mg/g, 0.12 mg/g, 4.08 mg/g, 0.72 mg/g and 5.06 mg/g respectively, in CB extract (Figure 6). The standard curve of each metabolite was showed good linearity at the tested concentration range (Table 2). The analytical method that we developed can be used as a quality control of CB extract.

**TABLE 2:** Calibration curves of metabolites.

Metabolite	Range (μg/ml, ppm)	Regression equation	*r* ^2^	LOD(μg/ml)	LOQ(μg/ml)
**Baicalin**	25.0–200.0	y = 0.8327x—2.4254	0.9991	0.0276	0.0838
**Geniposide**	25.0–200.0	y = 0.4116x—1.3258	0.9992	0.0560	0.1696
**Wogonoside**	25.0–200.0	y = 0.5609x—1.7040	0.9992	0.0410	0.1245
**Baicalein**	1.50–20.0	y = 1.6045x—0.4866	0.9998	0.0018	0.0056
**Hesperidine**	1.50–20.0	y = 0.2070x—0.0455	0.9998	0.0143	0.0435

LOD, 3.3 x σ/*S*. LOQ, 10 x σ/*S*.

σ is the standard deviation of the intercept from the regression equation and *S* is the slope of the calibration curve.

## 4 Discussion

CB is chiefly used to treat skin diseases due to its fever- and inflammation-relieving activities (Kim et al., 2019). Kim and Park et al. reported that the latter was exerted by modulating the activation of nuclear factor-κB and the phosphorylation of mitogen-activated protein kinase (Kim et al., 2017). Inflammation has been shown to be involved in the development of complex dysmetabolic processes, including fatty liver disease (Fricker et al., 2019; Petrescu et al., 2022). However, the therapeutic efficacy of CB has only been investigated in the context of skin inflammatory acne lesions or skin sensitivity. In this study, we hypothesized that CB could ameliorate fatty liver disease. Through both *in vivo* and *in vitro* experiments, we found that CB alleviated fatty liver disease by regulating the markers involved in lipid metabolism and activating AMPK.

Imbalances in lipid homeostasis, including the synthesis, degradation, and transport of the associated lipoprotein particles, result in abnormal lipid levels. Despite being essential components of the body, high levels of TGs, and cholesterol are risk factors for heart disease, stroke, dyslipidemia, and obesity. In addition, obesity chronically disturbs hepatic lipid metabolism, which may lead to metabolic comorbidities (Godoy-Matos et al., 2020). In this study, an animal model of HFD-induced fatty liver disease was employed to investigate the effect of CB on obesity-related hepatic lipid accumulation. HFD induced the major phenotypes of obesity, including body overweight, increased fat mass, and dyslipidemia, whereas CB significantly alleviated these symptoms (Figure 1). Meanwhile, CB did not affect food intake and gastrocnemius muscle mass, indicating that it could mitigate obesity without causing adverse effects like muscle atrophy.

HFD-associated obesity is very common in patients with non-alcoholic fatty liver disease, and is characterized by steatosis (Lian et al., 2020). We found that the livers of HFD-fed mice weighed more and accumulated lipids to abnormal levels. However, CB treatment significantly reduced lipid levels without inflicting liver damage (Figure 2). This phenomenon was confirmed in the human hepatocyte cell line HepG2 (Figure 4), confirming that CB can regulate abnormal lipid metabolism in hepatocytes.

The liver regulates lipid metabolism by orchestrating the synthesis of new fatty acids, their transport and redistribution to other tissues, and homeostasis (Du et al., 2022). Hepatic fat accumulates due to an imbalance between lipid acquisition and disposal through four major pathways: fatty acid uptake, *de novo* lipogenesis, mitochondrial fatty acid oxidation, and the export of lipids via lipoproteins (Ipsen et al., 2018). Therefore, regulating the accumulation of hepatic fat and related metabolic stages is fundamental to the prevention and treatment of fatty liver disease. In this study, lipid accumulation in HFD-fed mice was accompanied by expression changes in *CD36* and *FABP*, which are related to hepatocyte lipid uptake, *ACC*, *SREBP1*, *FAS*, and *LXRα*, which are related to TG synthesis, *SREBP2*, *HMGCoR*, *PCSK9*, *LDLR*, and *SR-B1*, which are related to cholesterol metabolism, and *CPT1α*, which is related to fatty acid oxidation. Lipid dysregulation orchestrated by these factors directly or indirectly contributes to fatty liver disease, especially the progression to obesity-associated hepatic steatosis. However, fatty liver symptoms were alleviated by CB in the HFD-fed mouse model and the FFA-treated hepatocyte model. Overall, CB not only inhibited lipogenesis and cholesterol synthesis but activated lipid oxidation both *in vitro* and *in vivo*.

AMPK is a well-known regulator of lipid metabolism. It inhibits the *de novo* synthesis of fatty acids and TGs by inhibiting SREBP1c, a transcription factor for the expression of lipogenic enzymes such as ACC and FAS (Jeon, 2016). AMPK induces the phosphorylation of ACC1 at Ser79 and ACC2 at Ser212, which in turn inhibits the conversion of acetyl-CoA to malonyl-CoA. This relieves the inhibition of CPT1, thereby activating fatty acid oxidation (Fullerton et al., 2013). Activated AMPK can also inhibit cholesterol synthesis by inducing the inhibitory phosphorylation of the rate-limiting enzyme 3-hydroxy-3-methylglutaryl-CoA reductase, thus downregulating the mevalonate synthesis pathway (Motoshima et al., 2006). We found that CB significantly increased the expression of phosphorylated AMPK in FFA-treated HepG2 cells (Figure 5), suggesting that CB mediated its effects via AMPK. These results suggest that AMPK plays a central role in regulating lipid metabolism by inhibiting lipogenesis and activating fatty acid oxidation. Being a natural AMPK agonist, CB is expected to regulate lipid metabolism, inflammation, and oxidative stress in hepatocytes (Fang et al., 2022).

In the context of fatty liver disease, the therapeutic properties of the individual botanical medicines constituting CB have been previously reported. The pharmacological activities of CB can be attributed to its botanical metabolites, such as baicalin, baicalein (flavonoid) from Scutellariae radix (Li et al., 2022a; Li et al., 2022b; Shi et al., 2022), geniposide (iridoid glycoside) from Gardeniae Fructus (Shen et al., 2020; Tang et al., 2022), liquiritigenin (flavanone), licochalcone A, isoliquiritigenin (chalcone) from *Glycyrrhizae Radix* et Rhizoma (Kim et al., 2011; Quan et al., 2013; Na et al., 2018), osthol (coumarin derivative) from Cnidii Rhizoma (Sun et al., 2010; Du et al., 2011), and berberine (alkaloid) from Coptidis Rhizoma (Sun et al., 2018). As expected, the individual botanical medicines act on various stages of non-alcoholic fatty liver disease via an AMPK-related signaling pathway, highlighting the prospect of AMPK as a potential target for treating metabolic disorders (Chen et al., 2018; Liou et al., 2019; Sun et al., 2020). Furthermore, these metabolites could be expected to exert a synergic effect in CB, but further research is required for validating this synergism and understanding the pharmacological mechanisms of each metabolite. Further examination of CB’s mechanism could develop insight into the applicability of CB to the associated metabolic diseases such as diabetes, obesity, and cardiovascular disease.

## 5 Conclusion

In conclusion, we demonstrated that CB effectively alleviated lipid accumulation in fatty liver disease. We uncovered a novel lipid-lowering mechanism wherein CB activated AMPK, thereby regulating the expression of transcription factors, its target enzymes, and β-oxidation-related genes. Based on the results of this study, CB could be a useful traditional medicine, and an effective candidate for preventing and/or treating fatty liver disease and associated metabolic diseases.

## Data Availability

The original contributions presented in the study are included in the article, further inquiries can be directed to the corresponding author.
